# Sensing by wireless reading Ag/AgCl redox conversion on RFID tag: universal, battery-less biosensor design

**DOI:** 10.1038/s41598-019-49245-3

**Published:** 2019-09-10

**Authors:** Nutcha Larpant, Anh Duc Pham, Atefeh Shafaat, Juan F. Gonzalez-Martinez, Javier Sotres, Johan Sjöholm, Wanida Laiwattanapaisal, Farnoush Faridbod, Mohammad Reza Ganjali, Thomas Arnebrant, Tautgirdas Ruzgas

**Affiliations:** 1 Department of Biomedical Science, Faculty of Health and Society, Malmö University, SE-205 06 Malmö, Sweden; 2 Biofilms - Research Center for Biointerfaces, Malmö University, SE-205 06 Malmö, Sweden; 3 Department of Clinical Chemistry, Faculty of Allied Health Sciences, Chulalongkorn University, Patumwan, Bangkok 10330 Thailand; 4 Center of Excellence in Electrochemistry, School of Chemistry, College of Science, University of Tehran, Tehran, Iran; 5 Pampett AB, 224 78 Lund, Sweden; 6 Biosensor Research Center, Endocrinology & Metabolism Molecular-Cellular Sciences Institute, Tehran University of Medical Sciences, Tehran, Iran

**Keywords:** Biosensors, Sensors

## Abstract

Massive integration of biosensors into design of Internet-of-Things (IoT) is vital for progress of healthcare. However, the integration of biosensors is challenging due to limited availability of battery-less biosensor designs. In this work, a combination of nanomaterials for wireless sensing of biological redox reactions is described. The design exploits silver nanoparticles (AgNPs) as part of the RFID tag antenna. We demonstrate that a redox enzyme, particularly, horseradish peroxidase (HRP), can convert AgNPs into AgCl in the presence of its substrate, hydrogen peroxide. This strongly changes the impedance of the tag. The presented example exploits gold nanoparticle (AuNP)-assisted electron transfer (ET) between AgNPs and HRP. We show that AuNP is a vital intermediate for establishing rapid ET between the enzyme and AgNPs. As an example, battery-less biosensor-RFID tag designs for H_2_O_2_ and glucose are demonstrated. Similar battery-less sensors can be constructed to sense redox reactions catalysed by other oxidoreductase enzymes, their combinations, bacteria or other biological and even non-biological catalysts. In this work, a fast and general route for converting a high number of redox reaction based sensors into battery-less sensor-RFID tags is described.

## Introduction

**Figure 1:**
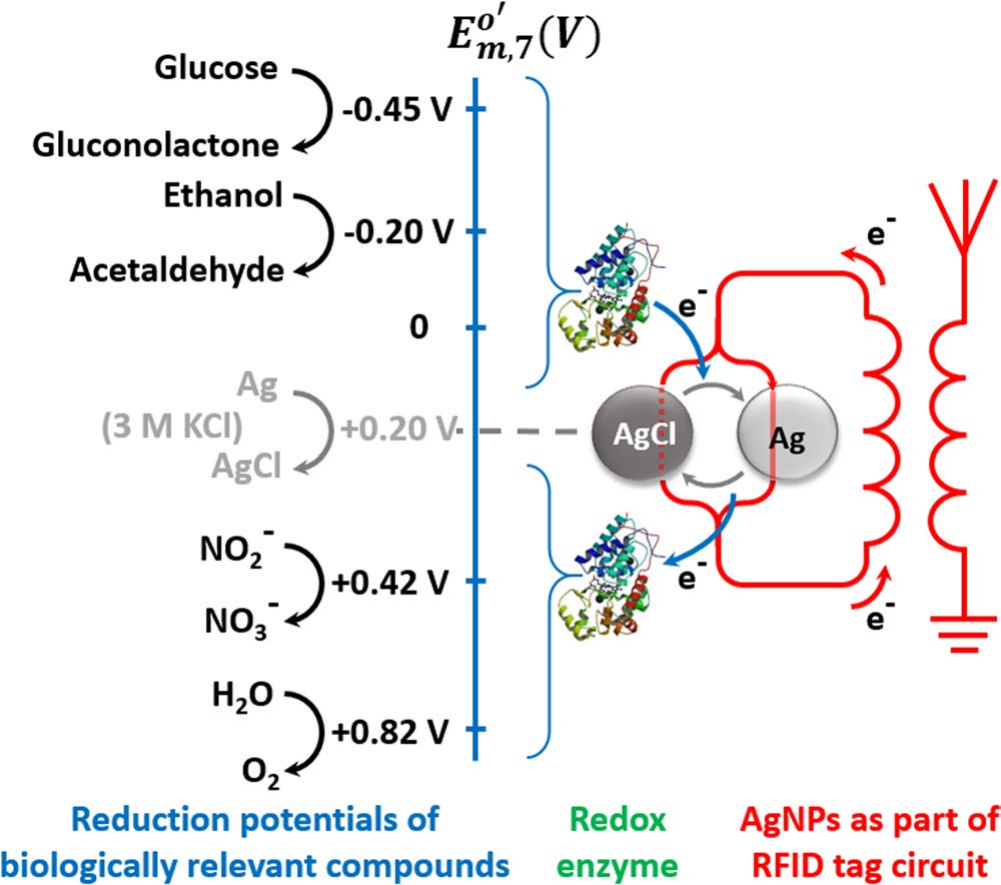
Conceptual representation of battery-less biosensor-RFID tag. AgNPs constitute a part of RFID tag antenna. An enzyme catalyzes oxidation or reduction of biologically relevant compound converting AgNPs to AgCl or AgCl-NPs to metallic Ag, respectively. This strongly modulates the impedance of the tag antenna. The impedance change is wirelessly monitored and is regarded as the biosensor response.

Since 1960s, redox enzyme based electrochemical biosensors, first described by Clark and Lyons^1^ and later improved by Updike and Hicks^2^ and Guilbault and Montalvo^3^, help millions of people in managing health disorders especially diabetes^4,5^. The usefulness of these biosensors is reflected by high earnings. Glucose biosensor market alone, according to Grand View Research Inc. estimate, will reach USD 31 billion by year 2022. This growth is followed by other redox enzyme based biosensors for cholesterol, lactate, glutamate, ascorbate, ketones, ethanol, etc. The principle of these biosensors is well-known and is based on oxidation or reduction of a substrate (analyte) catalyzed by a redox enzyme immobilized on the electrode^6^. The electron flow between substrate - enzyme - electrode is ensured by mediated (MET^7–10^) or direct electron transfer (DET^11–13^). For enabling and monitoring the electron flow a certain voltage, i.e., power source, is applied. To realize a battery-less and wireless monitoring of MET and DET reactions on RFID tag, we propose a biosensor-RFID tag design where a biological redox reaction drives Ag/AgCl redox conversion of AgNPs. The AgNPs in this design, constitute a part of the tag antenna. The universality of the design is due to the formal potential of Ag/AgCl redox reaction, being in the middle of reduction potential (E_m,7_^0^′) range of biologically relevant redox reactions (Fig. 1)^14^. Thus, oxidation of AgNPs to AgCl as well as reduction of AgCl-NPs to metallic AgNPs must be possible by a high number of biological redox reactions in chloride containing media.

Additionally, the proposed design takes advantage of the fact that the resistivity of the metallic Ag (1.6 * 10^−6^ Ω cm) and AgCl (3.5 * 10^9^ Ω cm) is 15-orders of magnitude different^15^. This difference is extremely important since it provides exceptionally strong modulation of the RFID-tag impedance by Ag/AgCl redox reaction and practically enables high sensitivity of wireless sensing in electrolyte solutions.

## Results and Discussion

**Figure 2:**
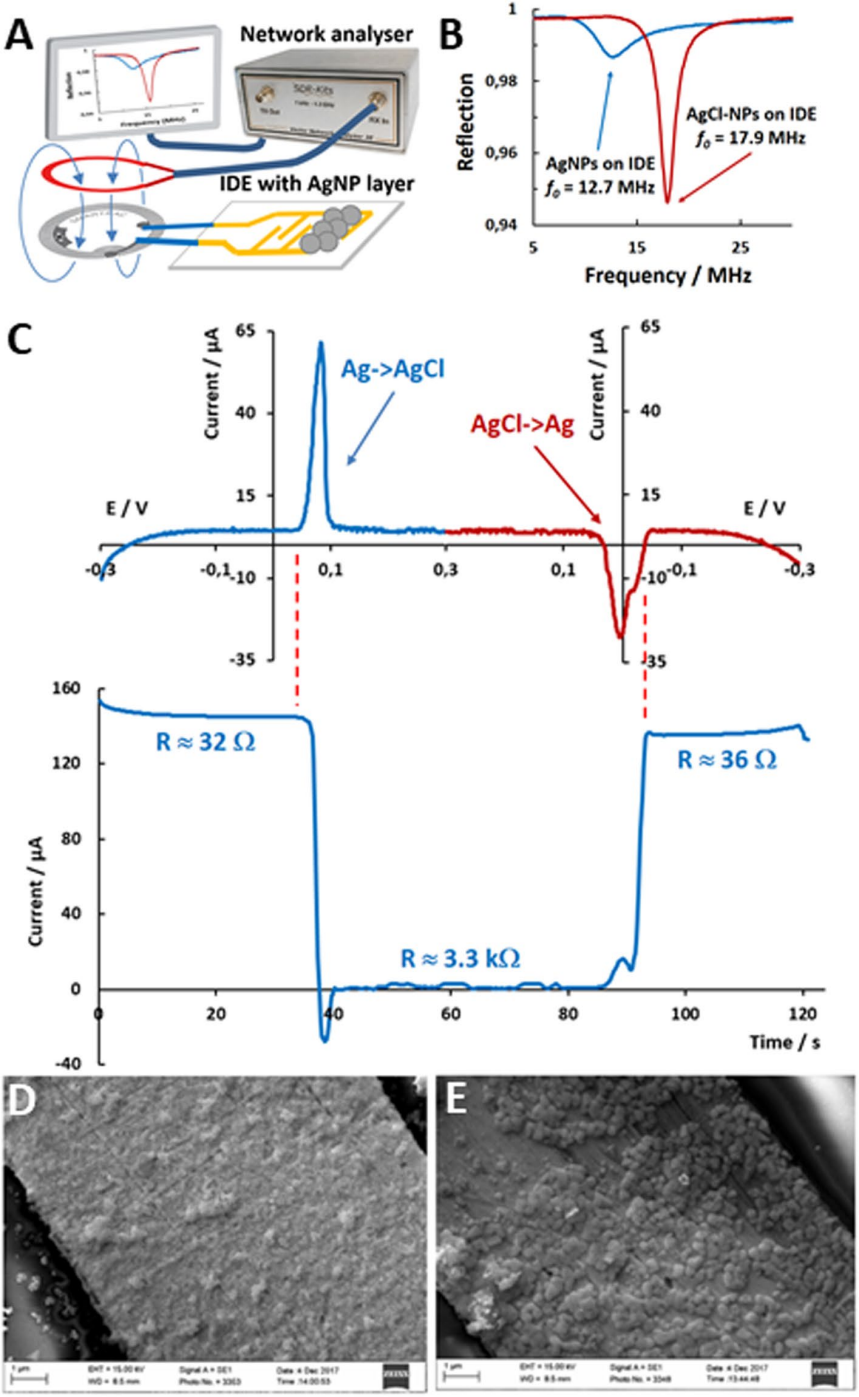
(**A**) The setup for the measurement of electromagnetic reflection from RFID tag containing the antenna-coupled IDE covered with a layer of AgNPs. (**B**) Reflection (S11) from the RFID tag containing IDE covered with a layer of metallic AgNPs or AgCl-NPs (IDE in air). (**C**) Simultaneous cyclic voltammetry (CV) and measurement of current between the fingers of the IDE. The CV measurements were done with IDE, covered with a layer of AgNPs, as a working electrode in PBS. Simultaneously with CV experiment, 5 mV DC voltage was applied between the fingers of the IDE and the resulting current was used to calculate the resistance of the IDE in PBS. (**D**,**E**) SEM image of AgNPs and AgCl-NPs on IDE, respectively.

In this work, the proof-of-concept demonstration of the battery-less RFID tag biosensor, based on Ag/AgCl conversion, was realized by connecting an interdigitated electrode (IDE), covered with a layer of AgNPs, into the tag antenna (Fig. 2A). Possibility of such IDE - tag antenna coupling has been described previously^16,17^. In this work, it was found that electromagnetic reflection from the antenna, Fig. 2B (reflection coefficient, S11) strongly depends on the oxidation state of the AgNPs. The tag containing IDE covered with a layer of metallic AgNPs showed resonance frequency at 12.7 MHz, while after electrochemical AgNP oxidation to AgCl-NPs and drying the resonance frequency shifted to 17.9 MHz. Simultaneously, resistance of the IDE changed from 32 Ω to more than 2 MΩ (these, RFID and resistance measurements were done with the IDE in air, see S2.3, supporting information). Electrochemical redox conversion of the AgNPs to AgCl-NPs (Fig. 2C, cyclic voltammogram) in phosphate buffer saline (PBS) showed that the resistance of the IDE in PBS changes by two orders of magnitude, i.e., from 32 Ω to 3.3 kΩ (the resistance calculated from the values of the current, that flow between the IDE fingers at 5 mV applied voltage, Fig. 2C (see S2.3 and Fig. S3.2 for details on the measurement setup). The experiments with AgNP modified IDE in air and in PBS prove high sensitivity of the RFID-IDE tag to Ag/AgCl redox reaction.

It is important that AgCl-NPs are practically insoluble^18,19^, they remain on the IDE surface (see SEM images, Fig. 2D,E) and, thus, can be electrochemically converted back to metallic AgNPs regaining low resistance of the IDE (36 Ω, Fig. 2C). The wireless RFID and the resistance measurements practically confirm that Ag/AgCl redox conversion in both directions, i.e., Ag −> AgCl and AgCl −> Ag, can be wirelessly monitored by the proposed RFID tag setup (Fig. 2A).

To demonstrate that redox enzymes can convert AgNPs to AgCl in the presence of an analyte, we used the enzyme horseradish peroxidase (HRP), which usually establishes facile DET on different electrode materials^12,20^. Considering thermodynamics, HRP in DET contact with AgNPs should be able to catalyze Ag −> AgCl conversion, Eq. 1.

1 $$A{g}_{NP}+{H}_{2}{O}_{2}+2{H}^{+}\mathop{\longrightarrow }\limits^{HRP,C{l}^{-}}AgC{l}_{NP}+2{H}_{2}O$$

**Figure 3:**
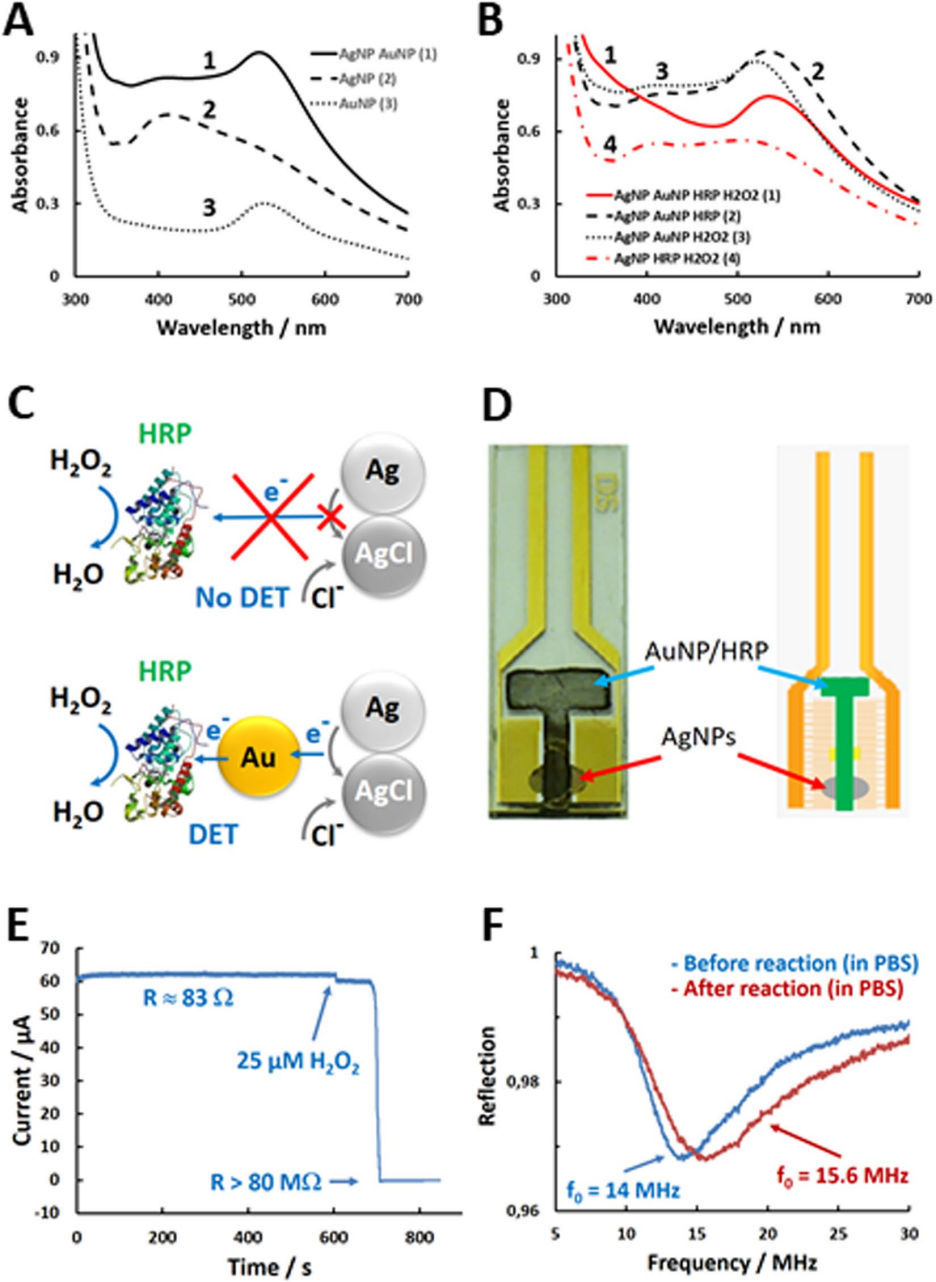
(**A**) Absorbance spectra of AuNPs, AgNPs and their mixture in PBS. (**B**) Absorbance spectra of AuNP, AgNP and HRP mixtures in PBS, in the presence and the absence of 0.1 mM H_2_O_2_. (**C**) Schematic presentation of AuNP-enabled enzymatic conversion of AgNPs to AgCl-NPs. (**D**) Photo and schematic presentation the electrode design hosting a layer of AgNPs short-circuiting the 2-mm gap between the two electrode areas. T-shaped area accommodates HRP/AuNP nanobiocompoud layer, which is in electrical contact with AgNPs. (**E**) The resistance of the electrode (made as in D) in PBS, before and after addition of 25 µM of H_2_O_2_. (**F**) RFID response (S11) before and after addition of H_2_O_2_. The AgNP and AuNP/HRP modified electrode was integrated into RFID tag and the reflection was recorded with the setup shown in Fig. 2A.

The question is, then, how to realize the electronic contact between HRP and AgNPs? Unfortunately, no convincing evidence has yet been obtained for DET between HRP and AgNPs. The absence of facile DET contact between HRP and AgNPs was additionally concluded from our spectrophotometric measurements of the mixture comprised of AgNPs and HRP in PBS containing 0.1 mM H_2_O_2_, since no change of the plasmonic adsorption from AgNPs was observed (λ_max_ ~ 400 nm, Fig. 3A,B) over prolonged period of time (more than 1 h). It is known that silver dissolution in the presence of chloride is very slow process and usually proceeds in tents of hours^21,22^. However, at the simultaneous presence of AuNPs, HRP and H_2_O_2_ the plasmonic feature of AgNPs (at ~400 nm, Fig. 3B) disappeared in few minutes, manifesting rapid AgNP conversion to AgCl-NPs (for details see supporting information, S2.5). The spectrophotometric study confirmed HRP/AuNP-facilitated AgNP oxidation by H_2_O_2_ to AgCl in PBS. It should be emphasized that for rapid AgNP conversion to AgCl, HRP/AuNP nanobiocomponent was required. The ET between the reaction components is schematically illustrated in Fig. 3C indicating the importance of AuNPs as an ET intermediate. Considering DET mechanism of HRP^20^ and interpreting the experimental results it can be concluded that the electrons must be abstracted from the metallic AgNP by AuNP and further transferred to the active site of HRP, where they reduce H_2_O_2_ to H_2_O. In order to integrate this sequence of redox reactions into RFID tag the components of the reaction were layered on IDE as shown in Fig. 3D. Then, the IDE was connected to the RFID tag as shown in Fig. 2A. To gain high sensitivity, the IDE was converted to two-electrodes separated by 2 mm gap by removing IDE fingers in the centre of the electrode (photo, Fig. 3D). The gap was covered (short-circuited) by a deposit of AgNPs. The AgNP deposit was, then, made in electrical contact with a T-shaped deposit of HRP/AuNP mixture as shown in Fig. 3D (for details see supporting information, S2.4).

HRP catalyzes AgNP oxidation to AgCl by H_2_O_2_ in PBS when the AgNP deposit is in electrical contact with the HRP/AuNP modified electrode (Fig. 3D) in PBS. This was initially confirmed by the direct current measurements between the electrode arms when the electrode was exposed to 25 µM H_2_O_2_. As can be seen in Fig. 3E, the electrode current changed from 60 µA to electrical noise level (approx. 50 pA), which corresponds to the resistance change from 83 Ω to higher than 80 MΩ. These experiments definitely prove rapid enzymatically driven AgNP oxidation to AgCl on the IDE at µM concentration of H_2_O_2_. Control experiment made without HRP or AuNPs showed that oxidation of AgNPs to AgCl is not possible at these low, sub-millimolar concentrations of H_2_O_2_. The study confirms that for rapid enzymatic AgNP oxidation to AgCl an ET pathway, AgNP −>AuNP −>HRP −>H_2_O_2_, must be established. In this particular example, HRP is in DET contact with AuNPs and the AuNPs are electronically connected to the AgNPs. Figure 3F demonstrates the electromagnetic reflection from the RFID tag before and after the H_2_O_2_/HRP/AuNP driven oxidation of AgNPs to AgCl. The tag with AgNP-short-circuited electrode, immersed in PBS, showed a resonance frequency at 14 MHz. After the addition of H_2_O_2_ into the PBS the resonance frequency shifted to 15.6 MHz (Fig. 3F). These results, for the first time, proves that Ag/AgCl redox conversion provides a universal route for plugging biological redox reactions into RFID tags. The design, thus, can serve a basis for developing wireless, battery-less biosensor-RFID tags based on redox enzymes.

To exemplify the universality, glucose oxidase was added on the surface of HRP/AuNP/AgNP nanobiocomponent-containing RFID tag. The RFID tag design became sensitive to glucose (see supporting information, S3.1). Similarly, any biosensor based on oxidase enzymes can be converted into battery-less biosensor-RFID tag using this universal approach. It should be pointed out that the presented biosensor-tag design might be better suited as a basis for a single-measurement and/or disposable biosensors; regeneration of AgNPs from AgCl, in most cases, leads to changed AgNP structures, which might affect the sensitivity of the sensor-tag.

**Figure 4:**
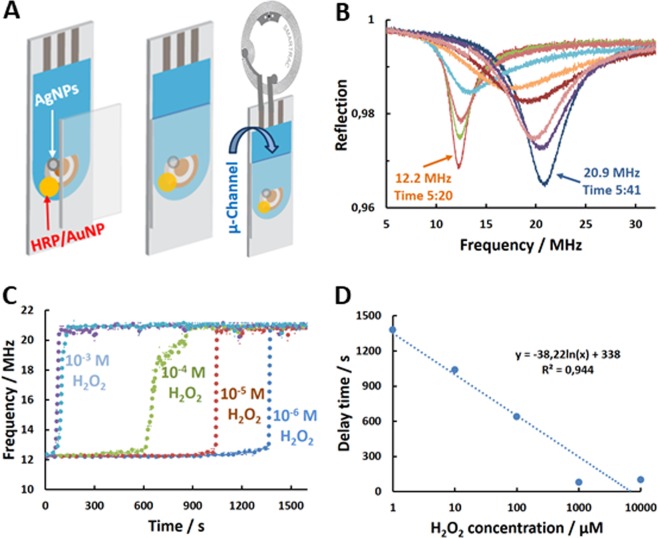
(**A**) Schematic representation of steps for making H_2_O_2_-biosensor RFID tag: (i) screen-printed electrode is modified with deposits of AgNPs and HRP/AuNP nanobiocomponent. The deposits are in electrical contact, however, only AgNPs short-circuit two adjacent electrodes on SPE, which are connected to the tag. (ii) The electrodes with AgNP and HRP/AuNP deposits are enclosed into µ-channel and (iii) coupled to a RFID tag. (**B**) Time dependent change of reflection from the H_2_O_2_-biosensor RFID tag after the microchannel was filled with PBS containing 1 mM H_2_O_2_. The time (min:s) is indicated for the first (right before the PBS in the µ-channel is replaced with PBS containing 1 mM H_2_O_2_) and the last (the moment when the reflection stabilizes at a new resonance frequency) reflection (S11) trace. (**C**) The resonance frequency of the tag vs time after the exposure of the electrode to specified concentrations of H_2_O_2_. (**D**) The dependence of the delay time of the resonance frequency shift from 12.2 to 21 MHz on H_2_O_2_ concentration in µ-channel.

In order to demonstrate that the battery-less H_2_O_2_ biosensor-RFID tag can discriminate different H_2_O_2_ concentrations, a cheaper, screen printed electrode (SPE) was modified with HRP/AuNP/AgNP nanobiocomponent as shown in Fig. 4A. In this construction, two electrodes on SPE were short-circuited with AgNP deposit, where HRP/AuNP deposit was placed in contact with only one of these two electrodes on SPE. Additionally, the electrode was enclosed into microfluidic channel for minimizing the effect of PBS conductivity on the RFID measurements (µ-channel minimizes high frequency short-circuiting by PBS electrolyte). The µ-channel was filled with PBS containing different concentrations of H_2_O_2_ and reflection from the tag was measured with network analyser (Fig. 1A). As can be seen in Fig. 4B the presence of H_2_O_2_ in PBS invokes gradual shifting of the reflection resonance frequency from 12.2 MHz to 21 MHz. It was found that the delay time required for the reflection, from the H_2_O_2_ biosensor-RFID tag, to reach 21 MHz depends on the H_2_O_2_ concentration (Fig. 4C,D). Our preliminary results (not shown) confirmed that the delay time is due to the need to transfer a defined amount of electricity, which is enough to convert deposited AgNPs to AgCl, i.e., subtract a defined amount of electrons from AgNPs and transfer them onto H_2_O_2_, according to Eq. 1. It is obvious that the delay time, and, thus, the sensitivity of the H_2_O_2_ biosensor-RFID tag can be regulated by changing the amount of deposited AgNPs. The observed shift of the resonance frequency from 12.2 MHz to 20.9 MHz is equivalent to 71% change, which must be regarded as a very high sensitivity of the proposed biosensor-tag to the redox reaction. Resonance frequency changes below 1% are usually considered as an acceptable wireless sensor responses^23^.

Similarly, to the H_2_O_2_ biosensor shown in Fig. 4 a wireless glucose biosensor was constructed and time needed to convert short-circuiting AgNP layer to AgCl was studied (S3.1, supporting information). Glucose dependent conversion of a highly electrically conductive layer of AgNPs (R = 19 ± 9 Ω, N = 5) to a non-conductive layer of AgCl was monitored by measuring a DC current between the two AgNP-short-circuited electrode arms (Fig. S6.2). After exposure of the biosensor to 1 mM of glucose, the DC current dropped from approximately 270 µA to a noise level in 700 s. Much quicker current drop (36 s) was obtained at 20 mM of glucose. The time required for converting the AgNPs to AgCl on glucose concentration (Fig. S6.2). This illustrates universal and general features of the proposed biosensor-RFID tag concept for sensing a broad range of redox reactions.

To assess the amount of Ag in form of AgNPs which, participates in the biosensor signal transducing reaction, the biosensor electrode was connected to potentiostate and the AgCl was electrochemically reduced back by running linear sweep voltammetry (see Fig. S6.2). It was found that 12.5 nmol (1.35 µg) of Ag were involved in the Ag/AgCl reaction. It can be suggested that shorter biosensor response times could obviously be achieved by lowering the amount of deposited AgNPs. This possibility will be addressed by us in the future.

In conclusion, we describe how combination of nanomaterials and enzymes can be exploited for making a wireless battery-less biosensor design based on RFID technology. The approach is universal due to the exploitation of AgNPs as part of RFID tag antenna. We show that biologically relevant redox reactions can drive Ag/AgCl redox chemistry on electrodes coupled to RFID tags, thus, strongly modulate the electromagnetic reflection from the tag. The fact that the formal potential of Ag/AgCl reaction is in the middle of the range of reduction potentials of biologically relevant reactions makes the proposed design “pluggable” to a vast number of biologically relevant reactions, i.e., the design must be highly universal. Additionally, the resistivity difference of metallic Ag and AgCl is close to 15-orders of magnitude, which enables development of highly sensitive biosensor-RFID tags. In this work, a proof-of-concept of biosensor-RFID tag has been shown by developing biosensors for H_2_O_2_ and glucose. We show that enzyme-nanoparticle combinations can be designed to drive AgNP oxidation to AgCl on RFID antenna. Since AgCl-NPs are practically insoluble, future work will be directed to demonstrate that similar biosensor-RFID tags can be developed based on enzymatic AgCl reduction to AgNPs. Generally, the discovery that rapid oxidation of AgNPs to AgCl can be catalyzed by enzymes with the assistance of, or mediated by, other, electrically conducting, nanomaterial, in this case AuNPs, is important not only for the development of wireless sensors but also for better understanding and control of AgNP fate in nature at low µM-concentrations of H_2_O_2_. Colloidal silver including AgNPs are broadly used as antimicrobial products and obviously come into contact with infection and inflammation site where H_2_O_2_ is present in µM concentrations^24,25^.

## Methods

### Synthesis of nanoparticles

AuNPs were synthesized by reduction of HAuCl_4_ with trisodium citrate. Briefly, 50 mL of aqueous HAuCl_4_ solution (1 mM) was prepared and heated to 80 °C under stirring. Then 10 mL of trisodium citrate (39 mM) was added to the above solution. The mixture was stirred and heated to 100 °C (~15 min) observing the color change from yellow to deep red. Then, the heating was stopped and the solution was left to reach room temperature in ~45 min under stirring. The gold nanoparticles were 5.4 nm in diameter and had −34 mV zeta potential determined by DLS. This dispersion of AuNPs was concentrated approx. 40 times by centrifugation. Final AuNP concentration was ~0.32 mM (0.34 mg/mL). For additional details, see S2.1 in supporting information.

AgNPs were prepared by reducing AgNO_3_ with trisodium citrate. Briefly, a mixture containing 2 mL of trisodium citrate (1 wt%), 1.5 mL of water, 0.5 mL of silver nitrate (1 wt%) and 1 mL of potassium chloride (8 mM) was prepared under stirring at room temperature. In parallel, 95 mL of water was heated to 70 °C and 100 µL of ascorbic acid (0.1 M) was added under stirring. After that the mixture of citrate, silver nitrate and potassium chloride was added to the ascorbic acid aqueous solution and continued heating to keep 70 °C temperature of the reaction mixture. The clear yellowish color of the solution developed in ~20 min. The solution was left under heating and stirring for additional 40 min. After that, AgNP dispersion was left to cool down to room temperature (no stirring). The dispersion was kept in fridge (+4 °C) for further application. The particle size and zeta potential were measured using DLS and were found to be equal to 11.8 nm and −20 mV, respectively. AgNP dispersion was concentrated approx. 100 times by centrifugation. For additional details, see S2.2 in supporting information.

### Preparation of AgNP modified IDE for cyclic voltammetry and resistance measurements

5 µL of 0.3 mM AgNP dispersion was dropped on the middle part of clean IDE and left to dry at room temperature for 1 h. The resistance measurement of AgNP-modified IDE during the electrochemical oxidation and reduction of AgNPs on AgNP-modified IDE was done by connecting two potentiostates. One of the potentiostates was used to run cyclic voltammetry between −0.3 and +0.3 V with AgNP modified IDE as a working electrode and Ag/AgCl and Pt wire as a reference and counter electrode, respectively. Another potentiostate measured current flow through the IDE at 5 mV applied voltage. The IDE electrode was in the measurement cell filled with PBS. The resistance of AgNP modified IDE was calculated using Ohm´s law, i.e., as a ratio of 5 mV to the measured current. For additional details, see S2.3 in supporting information.

Scanning electron microscopy images of NP modified electrode were obtained using Zeiss EVO LS10. The samples were recorded in electron back scattering mode at an accelerating voltage of 15 kV. The micrographs for all samples were recorded at magnifications of 1000.

### Preparation of NP modified electrodes to demonstrate HRP-catalyzed oxidation of AgNPs to AgCl

Central part (~2–3 mm) of fingers on IDE was removed using knife and emery paper. After that 0.2 mg/mL AuNP dispersion was drop casted several times (pipetted by 0.5 µL portions) to form a T-shaped AuNP electrode. Important, that the gold layer (T-shaped AuNP area on the electrode) was not allowed to electronically contact to the gold arms (and left fingers) of IDE. Then, the T-shape AuNP layer containing electrode was placed in a hot plate for drying at 65 °C for 10 min. After that 0.5 µL of 0.3 mM AgNPs was drop casted on each side of the gap between of the gold fingers of the IDE and the T-shaped AuNPs electrode. The electrode was left to dry at 65 °C for 10 min. This, AgNPs and AuNPs modified IDE was left to cool at room temperature and then 10 µL of 1 mg/ml HRP (solution in water) was dropped on T-shaped AuNP layer. The enzyme solution was kept on AuNP layer for 40 min at room temperature for HRP adsorption on AuNPs. After this the electrode was washed with water and the resulting AgNP/AuNP/HRP-modified IDE was used for electrical and RFID sensing of enzymatically catalyzed AgNP oxidation (AgNP −> AgCl) by H_2_O_2_ in PBS.

To demonstrate enzymatically catalyzed AgNP oxidation (AgNP −> AgCl) by H_2_O_2_ in PBS the HRP/AuNP/AgNP-modified IDE was immersed into PBS and connected to IVIUM potentiostate in two-electrode configuration. 5 mV potential was applied between the two electrode arms and current was recorded. After observing that the current is stable (for 10 min) a solution of H_2_O_2_ was pipetted into PBS giving 25 µM H_2_O_2_ concentration in the measurement cell. The solution in the measurement cell was stirred with magnetic stirrer during the entire experiment. For details, see S2.4 in supporting information.

### UV-VIS experiments to confirm AuNP enabled, enzymatically driven, AgNP oxidation to AgCl in solution

The absorbance spectra of solutions comprised of AgNPs, AuNPs, HRP, and H_2_O_2_ and their different mixtures were recorded by UV Vis Spectrophotometer. The test solution was prepared by mixing AuNPs and AgNPs, 0.2 µg/mL each. Then HRP solution was added to the particle mixture giving the final concentration of 10 µg/ml HRP. The mixture was incubated for 10 min at room temperature. After that H_2_O_2_ was added to give a final concentration of 0.1 mM H_2_O_2_ and the mixture was incubated for additional 10 min. The UV-VIS absorbance spectrum of the sample was recorded between 300 and 700 nm. To avoid NP precipitation all solutions were prepared in PBS diluted 10 times with water. For additional details, see S2.5 in supporting information.

### Modification of screen-printed electrodes

0.5 µL of concentrated AgNPs (~0.3 mM) was dropped to connect reference and working electrodes on screen printed electrode from Dropsens. 3 µL of concentrated AuNPs (~0.3 mM) was pipetted to connect counter and working electrodes. After drying, 5 µL of HRP (1 mg/mL) was dropped on the concentrated gold nanoparticles and left to dry at room temperature. After washing with water the electrode was enclosed into the microchannel with the thickness of 75 µm defined by a double sided tape, which was used to attach glass wall on the screen printed electrode. The working and the reference electrodes (i.e., the electrodes short-circuited by AgNPs) were then connected to RFID tag. The RFID signal was recorded continuously (each 4 seconds) after pipetting 50 µL of H_2_O_2_ solution into the microchannel. During all experiments the lower end of the microchannel was touching a bulk PBS solution in glass beaker. For details, see S2.6 in supporting information.

### RFID reader measurements and data processing

RFID signals from NFC tag connected to modified IDE or screen printed electrodes were recorded using a network analyzer DG8SAQ USB-Controlled VNWA 3EC coupled with a reading coil. This setup consists of a tag antenna, which was cut and connected to the interdigitated or screen-printed electrodes. For some photos and details, see S2.3 and S2.7 in supporting information.

## Supplementary information

Sensing by wireless reading Ag/AgCl redox conversion on RFID tag: universal, battery-less biosensor design
