# Supplementary material for: Sensing by wireless reading Ag/AgCl redox conversion on RFID tag: universal, battery-less biosensor design
Source: Sci Rep. 2019 Sep 10;9:12948. doi: 10.1038/s41598-019-49245-3 (PMC6736964; doi:10.1038/s41598-019-49245-3)
Supplement: Supplementary file 1 — Sensing by wireless reading Ag/AgCl redox conversion on RFID tag: universal, battery-less biosensor design [file 41598_2019_49245_MOESM1_ESM.docx]

**Supporting information**

**Sensing by wireless reading Ag/AgCl redox conversion on RFID tag: universal, battery-less biosensor design**

Nutcha Larpant^1,2,3^, Anh Duc Pham^1,2^, Atefeh Shafaat^1,2,4^, Juan F. Gonzalez-Martinez^1,2^, Javier Sotres^1,2^, Johan Sjöholm^5^, Wanida Laiwattanapaisal^3^, Farnoush Faridbod^4^, Mohammad Reza Ganjali^4,6^, Thomas Arnebrant^1,2^ and Tautgirdas Ruzgas^1,2^*

^1^Department of Biomedical Science, Faculty of Health and Society, Malmö University, SE-205 06, Malmö, Sweden. ^2^Biofilms - Research Center for Biointerfaces, Malmö University, SE-205 06, Malmö, Sweden. ^3^Department of Clinical Chemistry, Faculty of Allied Health Sciences, Chulalongkorn University, Patumwan, Bangkok, 10330, Thailand. ^4^Center of Excellence in Electrochemistry, School of Chemistry, College of Science, University of Tehran, Tehran, Iran. ^5^Pampett AB, 224 78, Lund, Sweden. ^6^Biosensor Research Center, Endocrinology & Metabolism Molecular-Cellular Sciences Institute, Tehran University of Medical Sciences, Tehran, Iran.

**S1. Materials and equipment**

*Materials.* Glucose, tablets of phosphate buffer saline (PBS), 35 % hydrogen peroxide solution, AgNO_3_, HAuCl_4_, tri-sodium citrate, sodium chloride, potassium chloride, glucose oxidase from *Aspergillus niger* (lyophilized, powder, ~ 200 U/mg), horseradish peroxidase (type VI, lyophilized powder, ≥ 250 units/mg), L-ascorbic acid, 25 wt% glutaraldehyde, multiwall carbon nanotubes were purchased from Sigma Aldrich. All solutions have been prepared by using deionized water purified by Milli-Q system (Merck Millipore, Billerica, USA) with resistivity of 18.2 Ω cm.

Gold interdigitated electrode (IDE) with 10 μm band-gap dimensions and gold screen printed electrodes (SPE) were from Dropsens, Llanera (Asturias), Spain. RFID tags (BullsEye NTAG213, with operation frequency 13.56 MHz) were purchased from RapidNFC Ltd, London, UK.

*Equipment.* Radio frequency identification (RFID) measurements were conducted using DG8SAQ Vector Network Analyzer v3E from SDR-Kits, Melksham, UK. Electrochemical experiments were conducted using IVIUM CompactStat, Ivium Technologies B.V., Eindhoven, The Netherlands. Scanning electron microscopy was done using Zeiss EVO LS10 from Carl Zeiss Microscopy GmbH, Jena, Germany. Nanoparticles were characterized by measurements using DLS/Zeta sizing system NICOMP 380 ZLS from PSSNYCOMP, Santa Barbara, California, USA.

**S2. Methods**

**S2.1. Synthesis of AuNPs and characterization by DLS**

Gold nanoparticles were synthesized by reduction of HAuCl_4_ with trisodium citrate following Turkevich method ^1^. Briefly, 50 mL of aqueous HAuCl_4_ solution (1 mM) were prepared and heated to 80^o^C under stirring. Then 10 mL of trisodium citrate (38.8 mM) were added to the above solution rapidly. The mixture was stirred and heated to 100^o^C (~15 min) observing that its color changed from yellow to deep red. Then, the heating was stopped and the solution was left to reach room temperature in ~45 min under stirring. The prepared gold nanoparticles were kept in the fridge (+4^o^C) over night and next day the particle size was assessed.

The particle size and zeta potential were determined by performing dynamic light scattering measurements using DLS/Zeta sizing system NICOMP. The scattering angle during the measurement was 90^o^. For all measurements, water was used as the dispersant with the index of refraction 1.333. The average run time for each DLS measurement was 5 min. The observed mean diameter (number weighting) and the average zeta potential of the AuNPs were 5.4 nm and -34 mV, respectively.

| 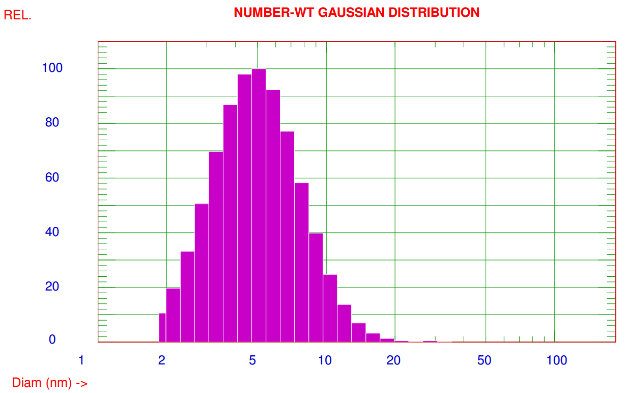 | 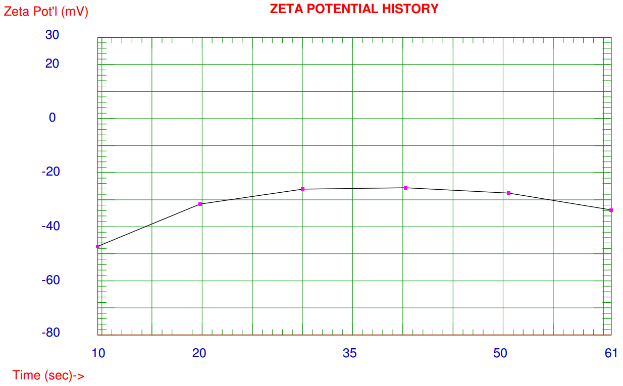 |
| --- | --- |
| **Fig. S1.** DLS measurement results: AuNP size distribution and their zeta potential. | |

Concentration of AuNPs after synthesis was ~ 8 nM (8.6 µg/mL) estimated from UV-Vis measurements ^2^. This dispersion of AuNPs was concentrated approx. 40 times by centrifugation and discarding the supernatant. Final AuNP concentration in concentrated AuNP dispersion was ~ 0.32 mM (0.34 mg/mL).

**S2.2. Synthesis of AgNPs and characterization by DLS**

Silver nanoparticles were prepared by reducing AgNO_3_ with trisodium citrate following recipe described by Li et al 2013 ^3^. Briefly, a mixture containing 2 mL of trisodium citrate (1 wt %), 1.5 mL of water, 0.5 mL of silver nitrate (1 wt %) and 1 mL of potassium chloride (8 mM) was prepared under stirring at room temperature and incubated (preferably in a dark) for ~5 min. In parallel 95 mL of water were heated to 70^o^C and 100 µL of ascorbic acid (0.1 M) were added under stirring for 1 min. After that the mixture of citrate, silver nitrate and potassium chloride was added to the ascorbic acid aqueous solution and continued heating to keep 70^o^C temperature of the reaction mixture. The clear yellowish color of the solution developed in ~20 min. The solution was left under heating and stirring for additional 40 min. After that, AgNP dispersion was left to cool down to room temperature (no stirring). The dispersion was kept in fridge (+4^o^C) until future use. The particle size and zeta potential were measured using DLS/Zeta sizing system NICOMP. The obtained mean diameter and average zeta potential of the AgNPs were 11.8 nm (number weighting) and -20 mV, respectively. AgNP dispersion was concentrated approx. 100 times by centrifugation and discarding the supernatant.

| 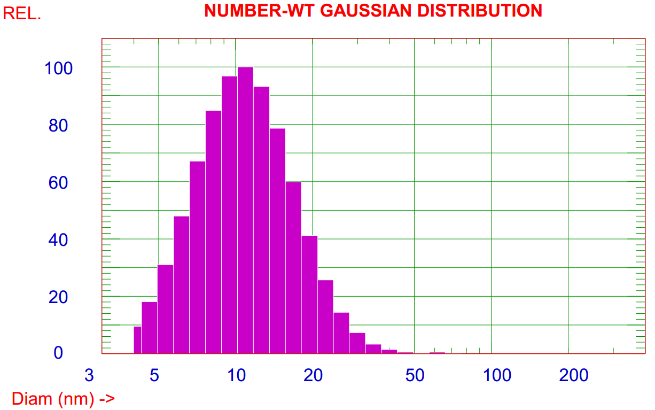 | 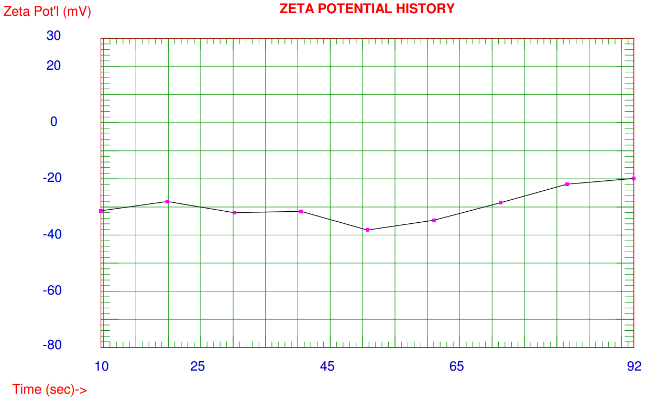 |
| --- | --- |
| **Fig. S2.** DLS measurement results: AgNP size distribution and their zeta potential. | |

**S2.3. Preparation of AgNP modified IDE electrodes, coupling to NFC tag, and cyclic voltammetry and resistance measurements**

Before the beginning of any modification process, IDEs were rinsed with 95 % ethanol, distilled water, blown with dry with N_2_, and finally plasma cleaned using Harrick Plasma Cleaner PDC32G, Harrick Scientific Corporation, NY (Ossining), for 5 min at the highest plasma intensity. Then, 5 µL of 0.3 mM AgNPs was dropped on the middle part of the IDE and left to dry at room temperature for 1 h. The IDE was connected to the RFID tag by using crocodile coupling (see photo shown in Fig. S3.1), which immediately allowed RFID measurements with the IDE in air.

| 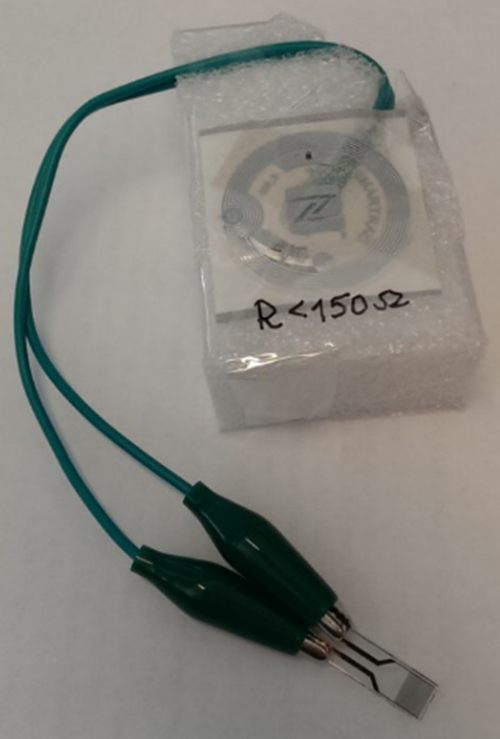 | **Fig. S3.1.** Photo of interdigitated electrode coupled to RFID tag. Some (~2 mm) path of the antenna was cut out and crocodile wires were glued using silver epoxy. Such a tag will also be read by NFC enabled mobile phone if resistance of IDE is below 150 Ω. |
| --- | --- |

The resistance measurement of AgNP-modified IDE during the electrochemical oxidation and reduction of AgNPs on AgNP-modified IDE (Fig. 2C) was done by connecting two potentiostats as shown in Fig. S3.2. One of the potentiostats was used to run cyclic voltammetry between -0.3 and +0.3 V and another to measure current flow through the IDE at 5 mV applied voltage. For conducting the measurements, the IDE electrode was immersed into the measurement cell filled with PBS. One side (arm) of the IDE was connected to IVIUM potentiostate as a working electrode. Additional Ag/AgCl (3M KCl) electrode was connected as a reference and platinum wire as counter electrodes, respectively. Together with CV the current through the IDE was measured with another IVIUM potentiostate and the current values were used to calculate the resistance of the AgNP-modified IDE (data are presented in Fig. 2C). The resistance was calculated using Ohm´s law, i.e., as a ratio of 5 mV to the measured current.


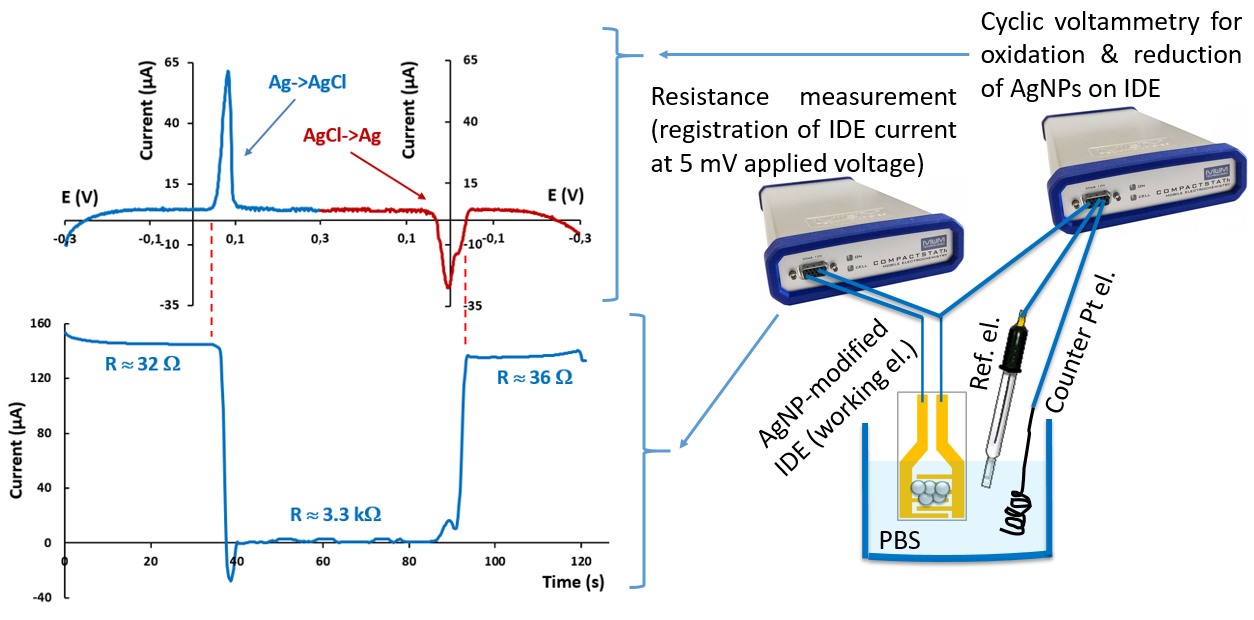


**Fig. S3.2.** Illustration of potentiostate-electrode connections for simultaneous measurements of current that flow through AgNP-modified IDE during electrochemical AgNP oxidation and reduction imposed by running cyclic voltammetry.

**S2.4. Preparation of NP modified electrodes to demonstrate HRP-catalyzed oxidation of AgNPs to AgCl (results presented in Fig. 3)**

Central part (~2-3 mm) of fingers on IDE was removed using knife and emery paper. Then the electrode was rinsed with 95% ethanol, distilled water, dried with N_2_, and cleaned by plasma cleaner for 5 min. After that 0.2 mg/mL AuNP dispersion was drop casted several times (pipetted by 0.5 µL portions) to form a T-shaped AuNP electrode. Important, that the gold layer (T-shaped AuNP area on the electrode) was not allowed to electronically contact to the gold arms (and left fingers) of IDE. Then, the T-shape AuNP layer containing electrode was placed in a hot plate for drying at 65^o^C for 10 min. After that 0.5 µL of 0.3 mM AgNPs was drop casted on each side of the gap between of the gold fingers of the IDE and the T-shaped AuNPs electrode. This was done to provide AgNP-based electrical connection between the T-shaped AuNP layer and gold fingers of the IDE. Then the electrode was left to dry at 65^o^C for 10 min. This, AgNPs and AuNPs modified IDE was left to cool at room temperature and then 10 µL of 1 mg/ml HRP (solution in water) was dropped on T-shaped AuNP layer. The enzyme solution was kept on AuNP layer for 40 min at room temperature for HRP adsorption on AuNPs. After this the electrode was washed with water and the resulting AgNP/AuNP/HRP-modified IDE was ready for RFID sensing of enzymatically catalyzed AgNP oxidation (AgNP -> AgCl) by H_2_O_2_ in PBS.

To demonstrate enzymatically catalyzed AgNP oxidation (AgNP -> AgCl) by H_2_O_2_ in PBS the HRP/AuNP/AgNP-modified IDE was immersed into PBS and connected to IVIUM potentiostate in two electrode configuration. 5 mV potential was applied between the two electrode arms and current was recorded (see Fig. 3E). After observing that the current is stable (for 10 min) a solution of H_2_O_2_ was pipetted into PBS giving 25 µM H_2_O_2_ concentration in the measurement cell. The addition of H_2_O_2_ resulted in drop of the current flowing between the arms of the electrode (see Fig. 3E). The current drop was interpreted as a loss of AgNP conductivity due to their enzymatically driven oxidation to AgCl. The solution in the measurement cell was stirred with magnetic stirrer during the entire experiment.

**S2.5. UV-VIS experiments to confirm AuNP enabled, enzymatically driven, AgNP oxidation to AgCl in solution (results presented in Fig. 3)**

To prove the AuNP-enabled enzymatic conversion of AgNPs to AgCl-NPs, the following experiment was conducted. The absorbance spectra of solutions comprised of AgNPs, AuNPs, HRP, and H_2_O_2_ and their different mixtures were recorded by UV-1800 UV Vis Spectrophotometer (Shimadzu). Table S1 summarizes the composition of samples. UV-Vis experiments were run as follows. The test solution containing all nano components was prepared by mixing AuNPs and AgNPs, 0.2 µg/mL each. Then HRP solution was added to the particle mixture giving the final concentration of 10 µg/ml HRP. The mixture was incubated for 10 min at room temperature. After that H_2_O_2_ was added to give a final concentration of 0.1 mM H_2_O_2_ and the mixture was incubated for additional 10 min. The UV-VIS absorbance spectrum of the sample was recorded between 300 and 700 nm. The control experiments (Table S1, control 1-3) and UV-VIS measurements were done with solutions excluding some components from the above mentioned mixture of AgNPs, AuNPs, HRP and H_2_O_2_. To avoid NP precipitation all solutions were prepared in PBS diluted 10 times with water.

**Table S1.** The composition of the test and control samples (No.1- 3). “+” represents the present of the component and “-” represents the absent of the component.

| Sample  in 10 times diluted PBS | AgNP  0. 2 µg/mL | AuNP  0.2 µg/mL | HRP  10 µg/mL | H_2_O_2_  0.1 mM |
| --- | --- | --- | --- | --- |
| The test sample | + | + | + | + |
| Control 1 (No H_2_O_2_) | + | + | + | - |
| Control 2 (No HRP) | + | + | - | + |
| Control 3 (No AuNP) | + | - | + | + |

UV-Vis absorbance spectra of gold and silver nanoparticle dispersions are given in Fig 3A and 3B. The AuNPs and AgNPs have UV-Vis absorbance peaks at ~525 nm and ~400 nm, respectively. Photos of some mixtures are presented in Fig. S4.

| 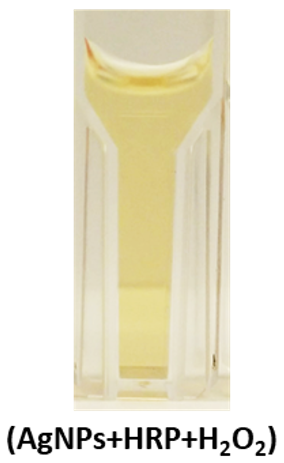 | 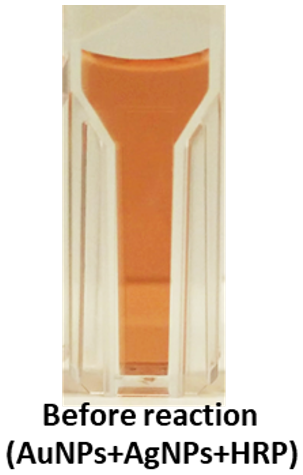 | 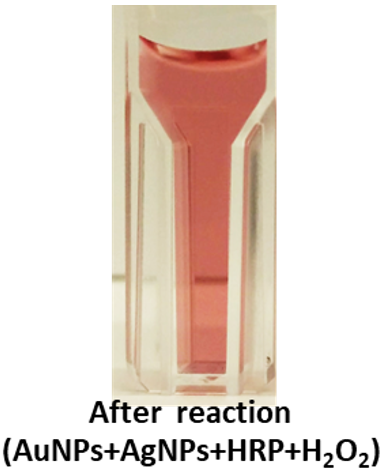 | **Fig S4.** Photos of AgNP, AuNP and HRP solutions in cuvettes (spectra in Fig. 3). Compositions of the mixtures are additionally specified in Table S1. |
| --- | --- | --- | --- |

**S2.6. Modification of screen-printed electrodes (results presented in Fig. 4)**

Briefly, 0.5 µL of concentrated AgNPs (~ 0.3 mM) was dropped to connect reference and working electrodes on screen printed electrode from Dropsens. 3 µL of concentrated AuNPs (~ 0.3 mM) was pipetted to connect counter and working electrodes. After drying, 5 µL of horseradish peroxidase (1 mg/mL) was dropped on the concentrated gold nanoparticles and left to dry at room temperature. After washing with water the electrode was enclosed into the microchannel with the thickness of 75 µm defined by a thickness of double sided tape, which was used to attach glass wall on the screen printed electrode. In this way, all modified electrodes were enclosed in 75 µm thick microchannel. The working and reference electrodes (short-circuited by AgNPs) were then connected to RFID tag as shown in Fig. 4. The RFID signal was recorded continuously (each 4 seconds) after pipetting 50 µL of H_2_O_2_ solution into the microchannel. During all experiments the lower end of the microchannel was touching a bulk PBS solution in glass beaker. This allowed surplus of the H_2_O_2_ solution, pipetted into microchannel, freely flow into the bulk solution, thus, not overloading the microchannel.

**S2.7. RFID reader measurements and data processing**

RFID signals were recorded using a network analyzer DG8SAQ USB-Controlled VNWA 3EC coupled with a reading coil. This setup consists of a tag antenna, which was cut and connected to the interdigitated or screen-printed electrodes as described in S2.3.

| 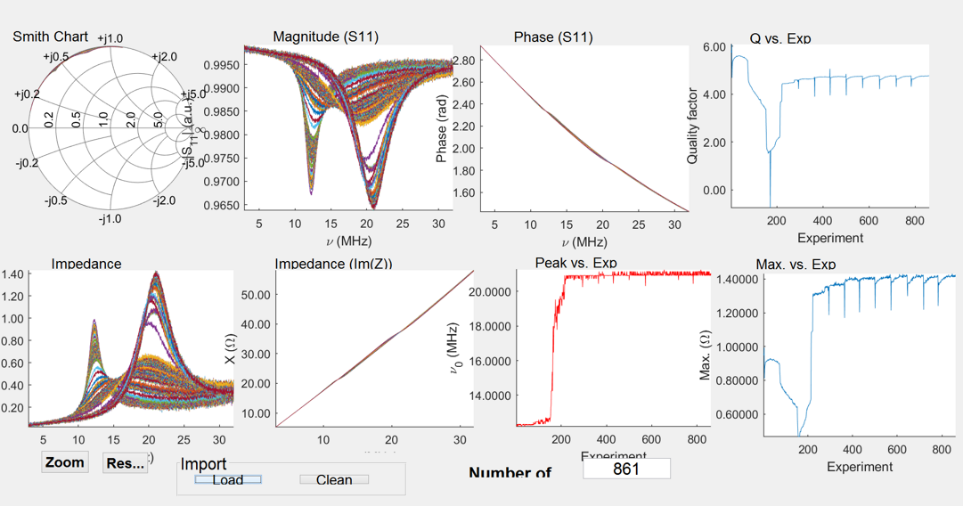 |
| --- |
| **Fig. S5.** Multiple measurement data presentation (screen-shot) obtained with SDR-kits DG85AQ Vector Network Analyzer 3E. The presentation includes also reflection parameter S11, which was used for studies of the tag-coupled biosensor electrodes. |

**S2.8. SEM equipment and measurements**

Scanning electron microscopy images were obtained using Zeiss EVO LS10. NP modified electrodes were placed on metallic sample holders and coated with silver glue paint at the edge of sample to secure electrical connection to the anode holder of SEM. The samples were recorded in electron back scatting mode at an accelerating voltage of 15 kV. The micrographs for all samples were recorded at magnifications of 1000.

**S2.9. Electrochemical equipment and measurements**

All the electrochemical measurements including cyclic voltammetry, linear sweep voltammetry and chronoamperometry were performed using IVIUM CompactStat. A platinum wire and an Ag/AgCl/KCl_sat_ were used as counter and reference electrodes, respectively. The interdigitated (IDE) and screen printed electrodes (SPE) from DropSens were used as working electrodes. Cyclic voltammetry and linear sweep voltammetry measurement were carried out over the potential range of -0.3 to 0.3 V at 10 mV/s. In chronoamperometry the current was recorded by applying 5 mV DC voltage during the measurement. Measuring the resistance of dry electrodes after modifications was done using a multimeter model EX542 (Extech Instruments, FLIR Systems, Inc., Waltham, MA).

**S3.0. Selection of nanomaterial with facile DET between HRP and the nanomaterial (potentiometric studies)**

To select suitable nanomaterial, enabling facile DET between HRP and the nanomaterial, the potentiometric experiments were carried out. Three-electrode electrochemical system was employed in these studies. Glassy carbon (GC) electrode (Ø= 3 mm), Pt wire, and Ag/AgCl (KCl saturated) were used as working, counter, and reference electrode, respectively. Briefly, 4 µL of 1 mg/ml HRP was dropped on electrode surface modified with different nanomaterial (see Table S2) and open circuit voltage (OCV) was measured in the PBS containing 0.1 mM H_2_O_2_. The procedures for preparing nanomaterial modified graphite electrode was as follows.

*Graphite flakes/HRP/Glutaraldehyde*. Graphite flakes and 4 µL of 1 mg/ml HRP was immobilized on GC electrode and 1 µL of 1%wt glutaraldehyde in water was added on top for protein cross-linking on the electrode surface.

*Multiwall carbon nanotube/Graphene oxide/HRP*. Briefly, 1 mg/mL grapheme oxide in water was mixed with 1.8 mg/ml multiwall carbon nanotubes. The combination was ultra-sonicated to reach homogeneity. Then 2 µL of multiwall carbon nanotube/graphene oxide mixture was applied on GC electrode and left drying in ambient air. After that 4 µL of 1 mg/ml HRP was immobilized on multiwall carbon nanotube/graphene oxide/GC electrode by simple physical adsorption. Specifically, HRP was let to dry during ~40 min. Finally, the electrode was washed with water.

*AuNP/HRP.* 2 µL of ~ 0.3 mg/mL AuNPs was drop casted on GC electrode and left to dry for 20 min. After that 4 µL of 1 mg/ml HRP was immobilized on AuNP modified electrode by letting the enzyme adsorb on electrode surface for 40 min. Finally, the electrode was washed with water.

*Multiwall carbon nanotube/graphene oxide /AuNP/HRP.* Firstly, 2 µL of ~ 0.3 mg/mL AuNP was drop casted on GC electrode and left drying for 20 min after that 2 µL of multiwall carbon nanotube/graphene oxide mixture was applied on the electrode and left drying in ambient air. Lastly, 4 µL of 1 mg/ml HRP was immobilized on multiwall carbon nanotube/graphene oxide /AuNP/GC electrode by letting the enzyme adsorb on electrode surface for 40 min. The modified electrode was then washed with water and transferred to electrochemical cell for OCV measurement.

**Table S2.** Open circuit voltage (OCV) measured with nanomaterial - HRP modified electrodes in PBS containing 0.1 mM H_2_O_2_.

| Materials | OCV (V) vs Ag/AgCl/KCl(sat) |
| --- | --- |
| 1. Glassy carbon (GC)/HRP/Glutaraldehyde  2. Multiwall carbon nanotube/Graphene oxide/HRP  3. AuNP/HRP  4. Multiwall carbon nanotube/graphene oxide /AuNPs/HRP | 0.0014  0.075  0.48  0.43 |

As can be seen from Table S2, AuNP/HRP combination gives the most positive OCV indicating the most facile DET between the HRP enzyme and the nanomaterial ^4^.

**S3.1. RFID-tag based glucose biosensor: proof-of-concept**

To exemplify the universality of battery-less biosensor-RFID tags based on redox enzymes, glucose sensing was assessed. The preparation of NP modified electrode was similar to (S2.4) with few modifications. Briefly, 2 µL of ~0.3 mg/mL AuNPs was drop casted 10 times (20 µL in total) to make a T-shape area (front), square-shape area (back) electrode and the edge connecting T-shape front and the square back covered with dense AuNP layer. This ensured high surface area of AuNP-comprised electrode (Fig. S6.1). Then, 0.5 µL of ~ 0.3 mM AgNPs was pipetted on the gaps between T-shaped AuNP layer and the fingers of IDE (as shown in photo, Fig. 3 and sketched in Fig. S6.1), short-circuiting the arms of the electrode. Then the electrode was kept just above the hot plate for drying during 10 min. After cooling at room temperature, 20 µL of HRP (1 mg/mL) was applied on entire AuNP covered area resulting into HRP-modified AuNP-comprised electrode (HRP/AuNP electrode). After 40 min, 20 µL of glucose oxidase (2 mg/mL) was applied on the top of the HRP/AuNP electrode and let to adsorb for 40 min. In total five electrodes have been prepared, they have been used for one measurement, thus, data statistics account these five measurements. The response of the described glucose sensors (Fig. S6.1) was assessed in different glucose concentrations by measuring the time needed to convert AgNPs to AgCl. The conversion was monitored by registering current which flow between the arms of the electrode at 5 mV applied voltage or by recording an electromagnetic reflection curves. The data are summarized in Fig. S6.2. To appreciate the results it should be noted that the mechanism of GOx/HRP/AuNP glucose biosensor is based on glucose oxidation catalyzed by GOx. This reaction produces H_2_O_2_. The H_2_O_2_ then oxidizes AgNPs to AgCl catalyzed by HRP being in direct electron transfer contact with AuNPs (see main text, Eq. 1, Fig. 3). The deposition of nanoparticles is made in such a way that AuNPs are in electrical contact with AgNP deposit (Fig. S6.1).

| 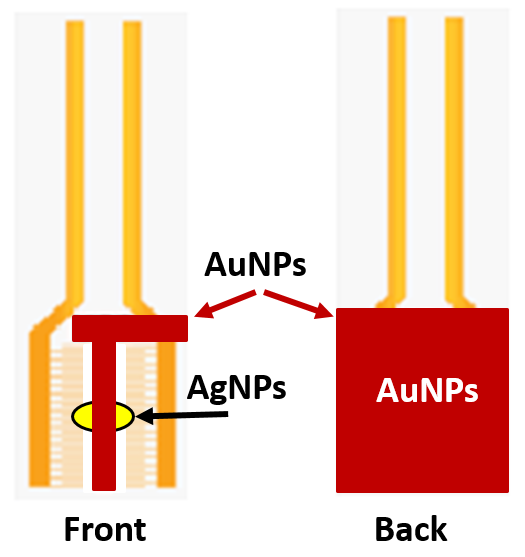 | 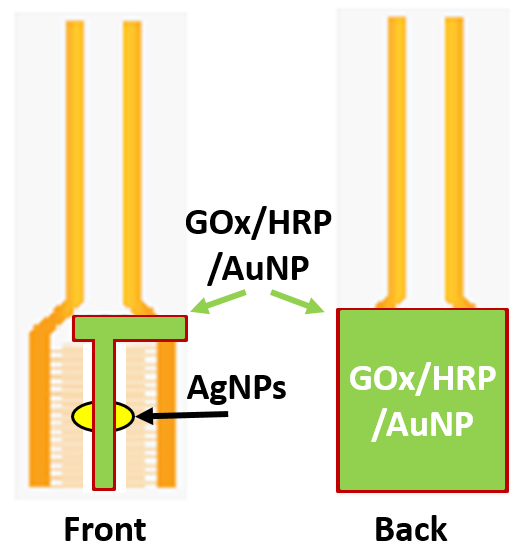 |
| --- | --- |
| **Fig. S6.1.** Schematic illustration of nanoparticle modified electrode for RFID tag based glucose sensing. After deposition of Au and Ag nanoparticles, next, HRP was adsorbed on front and back sides of AuNP comprised electrode. After that, GOx was adsorbed on top of HRP/AuNP electrode area. Both side of the electrode had electrical connection by AuNP deposit going through the electrode edge. The arms of the biosensor electrode were short-circuited by AgNP deposit (indicated by yellow area). | |

*Electrochemical measurement.* The above described glucose sensor (Fig. S6.1) was immersed into PBS, agitated by magnetic stirrer. Potential of 5 mV was applied between the arms of the biosensor electrode and current was recorded. As can be seen from Fig. S6.2 (see measurement setup and current-time dependence) the current decreased to zero (a noise level) after addition of glucose into the cell. The current decrease is due to the production of H_2_O_2_ during glucose oxidation with the following H_2_O_2_ based AgNP oxidation to AgCl. Conversion of AgNPs to AgCl result in increased resistance between the arms of the biosensor, thus, dropping current. As can be seen from Fig. S6.2, the time needed to reach zero current (complete loss of conductivity between the arms of the biosensor electrode) is proportional to the glucose concentration (Fig. S6.2, time-concentration dependence). To determine how much Ag is involved in the reaction, the biosensor electrode, after experiment with glucose, was connected to three electrode potentiostate. The AgCl on the biosensor electrode was electrochemically reduced to Ag by running linear sweep voltammetry (Fig. S6.2, the insert, potential scan from 0.2 to -0.2 V). The current-potential dependence is a typical linear sweep voltammogram reflecting AgCl reduction to Ag. Integral of this curve gives a charge and allows calculation of total AgCl involved in the reaction. Total charge was equal to 1.20±0.21 mC (average of five similarly prepared electrodes), which corresponds to 12.5 nmol (1.34 µg) of Ag. Obviously, this is the amount of Ag deposited in form of AgNPs on the biosensor electrode. Higher amount of Ag on the electrode requires longer time of its conversion to AgCl at the same concentration of glucose (this has been noticed in preliminary experiments, but not yet systematically studied). Thus, changing the amount of AgNPs on the electrode should allow tuning the biosensor sensitivity (this issue will be studied in the future).

| 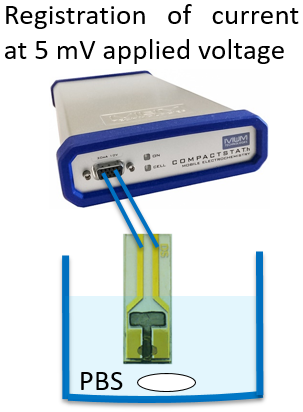 | 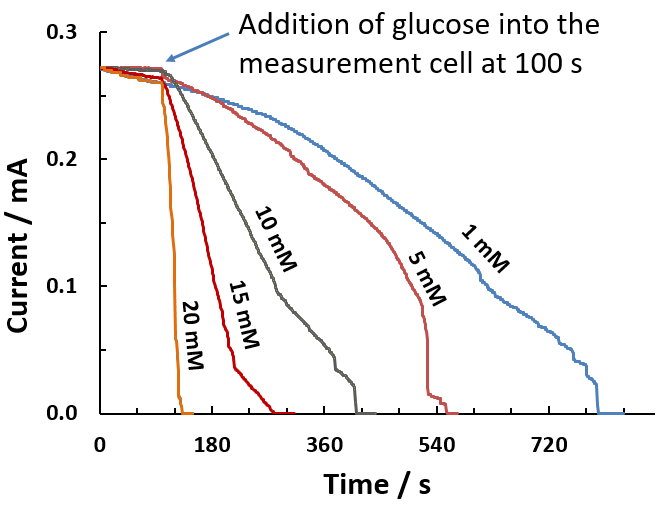 |
| --- | --- |
| 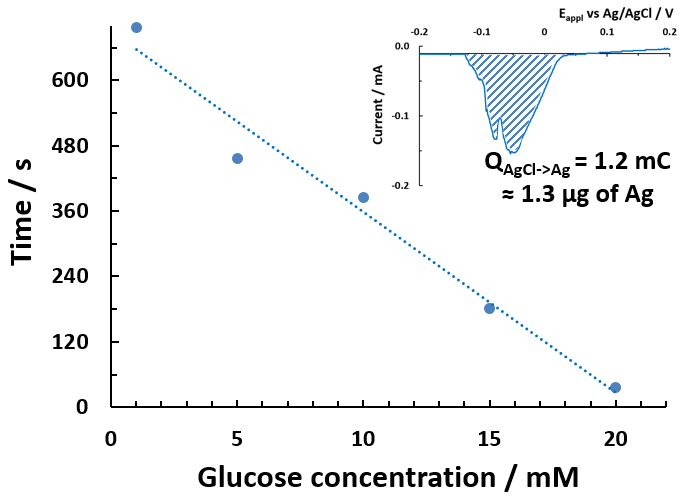 | 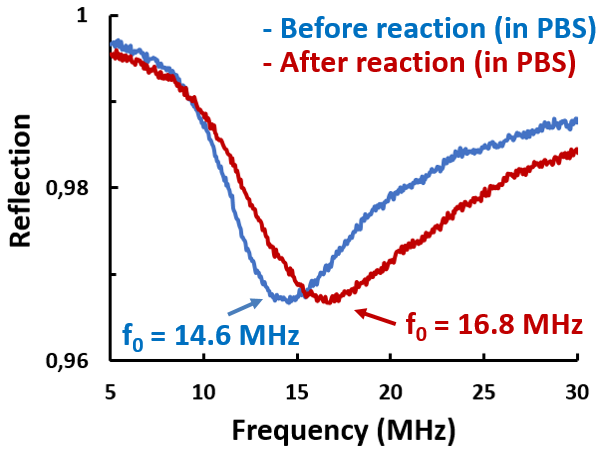 |
| **Fig. S6.2.** Measurement setup comprised of glucose biosensor connected to a potentiostate. Current is recorded at 5 mV applied between the arm of the biosensor electrode. Current-time dependence illustrate the current decay to zero after the addition of glucose into PBS solution (glucose concentration in PBS is specified for each curve). Time-concentration dependence illustrate that the decay time depends on glucose concentration spanning from 700 s to 36 s at 1 and 20 mM of glucose, respectively. The RFID response (corrected S11 magnitude) of glucose biosensor before and after addition of 1 mM glucose. The RFID-tag connected to the arms of the biosensor electrode similarly as shown in Fig. S3.1. During RFID measurement the biosensor is in PBS. | |

*RFID measurement.* The described glucose biosensor (Fig. S6.1) was connected to the RFID tag (as in Fig. S3.1) and the reflection from the tag was recorded using SDR-kits DG85AQ Vector Network Analyzer 3E. The RFID response (corrected S11 magnitude) and the resonance frequency shift before and after addition of 1 mM glucose are shown in Fig. S6.2 and summarized in Table S3. µ-channel in this case was not used. It was noticed that the sensitivity of RFID measurement is sufficient if the AgNP deposit is longer than 2 mm. This was achieved by scratching gold fingers, i.e., making 6 mm gap, on the central part of the IDE (Fig. S6.1).

**Table S3.** The resonance frequency shift of the tag connected to the glucose biosensor (Fig. S6.1). The frequency shift is due to the oxidation of glucose and generation of H_2_O_2_, which drive oxidation of AgNPs to AgCl as explained in the paragraph under “*Electrochemical measurement*”.

| f_0_ (MHz),  before addition of glucose | f_0_ (MHz),  after addition of 1 mM of glucose | ∆f_0_ (MHz) |
| --- | --- | --- |
| 14.56 | 16.75 | 2.19 |

**REFERENCES**

1. Turkevich J, Stevenson PC, J. H. A study of the nucleation and growth processes in the synthesis of colloidal gold. *J Discuss Faraday Soc* 1951, **11:** 55-75.

2. Haiss W, Thanh NTK, Aveyard J, Fernig DG. Determination of size and concentration of gold nanoparticles from UV−Vis spectra. *Anal Chem* 2007, **79**(11)**:** 4215-4221.

3. Li H, Xia H, Wang D, Tao X. Simple Synthesis of Monodisperse, Quasi-spherical, CitrateStabilized Silver Nanocrystals in Water. *Langmuir* 2013, **29:** 5074-5079.

4. Ruzgas T, Csöregi E, Emnéus J, Gorton L, Marko-Varga G. Peroxidase-modified electrodes: Fundamentals and application. *Anal Chim Acta* 1996, **330:** 123-138.
